# Effects of multicomponent exercise on global cognitive function, executive function, and brain-derived neurotrophic factors in older adults with mild cognitive impairment: a systematic review and three-level meta-analysis

**DOI:** 10.3389/fnagi.2026.1921425

**Published:** 2026-09-21

**Authors:** Pengfei Yang, Yongjie Liu, Yiyi Duan, Nguyen Manh Cuong, Yun Bi

**Affiliations:** 1 Department of Physical Education, Shanghai Dianji University, Shanghai, China; 2 Jiaxing University, Jiaxing, China; 3 Shanghai University of Sport, Shanghai, China; 4 Faculty of Basic and Fundamental Sciences, Danang Sport University, Da Nang, Vietnam; 5 Physical Education Department, Public Basic Courses Division, Shanghai Conservatory of Music, Shanghai, China

**Keywords:** multicomponent exercise, older adults with mild cognitive impairment, global cognitive function, executive function, brain-derived neurotrophic factor, meta-analysis, randomized controlled trial

## Abstract

**Objective:**

To systematically evaluate the effects of multicomponent exercise on global cognitive function, executive function, and brain-derived neurotrophic factor in older adults with mild cognitive impairment using a three-level meta-analysis.

**Methods:**

Web of Science, Cochrane Library, Embase, EBSCOhost, PubMed, CNKI, Wanfang, and Weipu were systematically searched from inception to September 15, 2025, to identify randomized controlled trials (RCTs) examining multicomponent exercise interventions in older adults with mild cognitive impairment. Methodological quality was assessed using the PEDro scale, and the quality of evidence was evaluated using the GRADE system. Meta-analysis was conducted using a three-level random-effects model in R software, and publication bias was examined. Effect sizes were estimated using Hedges’ *g* with 95% confidence intervals (CIs), and 95% prediction intervals (PIs) were calculated to determine the expected range of effect sizes in future comparable studies. Funnel plots and Egger’s regression test were used to assess publication bias.

**Results:**

A total of 10 RCTs comprising 623 older adults with MCI from community settings, hospitals, and nursing homes were included. The interventions consisted primarily of multicomponent exercise at moderate intensity, with session durations of 30–90 min, frequencies of 2–5 sessions per week, and intervention periods of 8–48 weeks. Control groups received usual care or health education. Most studies reported no exercise-related adverse events. The mean PEDro score of the included studies was 6.0. The three-level meta-analysis revealed that multicomponent exercise significantly improved global cognitive function (*g* = 1.063, 95% CI [0.355, 1.771], *p* = 0.007, 95% PI [−1.141, 3.267]) and executive function (*g* = 0.372, 95% CI [0.140, 0.604], *p* = 0.005, 95% PI [0.140, 0.604]) in older adults with MCI. BDNF was assessed in only two studies, with conflicting findings, and its effects remain inconclusive. Exploratory subgroup analyses revealed that none of the moderator variables reached statistical significance: exercise duration [*F*(1,9) = 0.127, *p* = 0.730], exercise frequency [*F*(1,10) = 3.421, *p* = 0.094], intervention duration [*F*(1,10) = 0.0002, *p* = 0.988], and measurement tools [*F*(2,9) = 0.882, *p* = 0.447] all showed no significant moderating effects. Owing to the limited number of effect sizes in certain categories (*k* ≤ 2), interaction tests were not performed for exercise modality, and results across groups were presented descriptively. Descriptive analyses showed that the combinations of “aerobic + strength + balance + coordination + agility” training (*k* = 2, *g* = 2.109, 95% CI [0.816, 3.402], *p* = 0.006) and “aerobic + strength + flexibility” training (*k* = 2, *g* = 2.039, 95% CI [0.772, 3.307], *p* = 0.007) yielded large effect sizes. Interventions with session durations of 30–60 min improved global cognitive function (*g* = 1.236, 95% CI [0.151, 2.321], *p* = 0.030), as did those with frequencies of 4–5 sessions per week (*g* = 1.772, 95% CI [0.714, 2.830], *p* = 0.004). Assessments using both the MoCA (*g* = 1.337, 95% CI [0.235, 2.439], *p* = 0.023) and the MMSE (*g* = 1.054, 95% CI [0.214, 1.895], *p* = 0.020) supported the positive effects of multicomponent exercise on global cognition. With respect to executive function subdomains, cognitive flexibility was significantly improved (*g* = 0.437, 95% CI [0.112, 0.761], *p* = 0.014), whereas working memory and inhibitory control did not reach statistical significance.

**Discussion:**

The overall methodological quality of the included studies was moderate; however, most studies did not implement allocation concealment or fully report blinding procedures, indicating a certain risk of bias. Tests for publication bias did not indicate significant bias. GRADE assessment showed that the quality of evidence was low for global cognitive function and moderate for executive function. Multicomponent exercise exerts positive effects on global cognitive function and executive function in older adults with MCI, whereas its effects on BDNF remain inconclusive. Future studies with more rigorous designs and adequate sample sizes are warranted to promote the personalized application of multicomponent exercise in the MCI population.

**Systematic review registration:**

This study was registered in PROSPERO (No. CRD420251164302; https://www.crd.york.ac.uk/).

## 1 Introduction

In the context of the continuing global population aging, data from the World Health Organization (WHO) indicate that the proportion of individuals aged 60 and above is rapidly increasing, and it is projected to account for 22% of the global population by 2050 (Bajwa et al., 2019). Cognitive impairment is one of the most common health issues among the elderly, with an overall prevalence of 33.59% (He et al., 2023), Alzheimer’s disease (AD) has become the third leading cause of death, following cardiovascular and cerebrovascular diseases and malignant tumors (Liu et al., 2017). Mild Cognitive Impairment (MCI) is a clinical transitional state between normal cognitive aging and AD, characterized by unique bidirectional outcomes: on one hand, it is a crucial precursor to AD, with an annual conversion rate of approximately 15% (Rao et al., 2018); on the other hand, there is a possibility of cognitive function reversal in over 30% of cases (Han et al., 2022). Unlike the irreversible neuronal damage caused by AD, studies have confirmed that early intervention can effectively delay or even prevent the progression of MCI to AD (Livingston et al., 2017). Therefore, implementing timely and effective intervention strategies during the MCI stage is of significant clinical and public health importance for delaying cognitive decline, reducing the burden on social care, and improving individual quality of life.

Older adults with MCI experience a decline in overall cognitive function, with the most significant and clinically specific decline observed in executive function (Marshall et al., 2011). Executive function, as a collective term for a series of higher-order cognitive control processes, consists of core components such as working memory, cognitive flexibility, and inhibitory control, among other key subdomains. These functions collectively form the foundation for goal-directed behavior and directly influence abilities such as planning, decision-making, problem-solving, and environmental adaptation (Miyake et al., 2000). At the neurobiological level, the pathological mechanisms of MCI are closely associated with impaired synaptic plasticity and insufficient neurotrophic support. Studies show that BDNF levels in the peripheral blood of MCI older adults are significantly reduced, which may be one of the key molecular bases mediating cognitive decline, particularly executive function impairment (Kim et al., 2025; Ward et al., 2015). Therefore, identifying intervention strategies that can effectively improve overall cognitive function, executive function, and regulate BDNF levels is of significant importance for delaying the progression of MCI.

Numerous studies have confirmed that exercise interventions can effectively promote cognitive function in older adults (Livingston et al., 2017). Some studies have found that exercise interventions can not only delay the cognitive decline process in MCI patients but also produce positive therapeutic effects (Tarumi et al., 2019; Chen et al., 2020); However, other research has shown that exercise interventions lasting 12 to 24 months failed to significantly improve cognitive performance in this population (van Uffelen et al., 2007; Sink et al., 2015). Among various exercise programs, multicomponent exercise (defined as a mixed exercise approach that includes two or more types of exercise, such as aerobic exercise, strength/resistance training, balance/coordination training, and flexibility training) can induce more new neurons due to its diverse and rich exercise environment, thereby improving cognitive function in MCI older adults (Kuehn, 2015; Huang et al., 2022). Its potential mechanisms may involve increasing cerebral blood flow, elevating peripheral BDNF levels, promoting neurogenesis, enhancing synaptic plasticity, and delaying brain structural atrophy, all contributing to cognitive protection (Gligoroska and Manchevska, 2012; Hyman et al., 1991). The exact effects of multicomponent exercise on overall cognitive function, executive function, and BDNF in MCI older adults still require further verification.

Accordingly, this study comprehensively collected published randomized controlled trials and employed a three-level meta-analytic approach. A three-level model was used to account for multiple effect sizes within each study, thereby maximizing the utilization of original data and optimizing statistical power (Xu et al., 2024). The study systematically evaluated the effects of multicomponent exercise on global cognitive function, executive function, and brain-derived neurotrophic factors in older adults with mild cognitive impairment. In addition, subgroup analyses were conducted based on exercise modality, exercise duration per session, exercise frequency, and intervention duration to examine whether components of the exercise prescription serve as potential moderators of cognitive outcomes. This study aims to provide evidence-based exercise recommendations for delaying and improving cognitive decline in older adults and to inform clinical practice.

## 2 Methods

This study followed the Preferred Reporting Items for Systematic Reviews and Meta-Analyses (PRISMA) guidelines (Page et al., 2021) and has been registered in the International Prospective Register of Systematic Reviews (PROSPERO) (Registration No. CRD420251164302).

### 2.1 Literature search strategy

Two researchers independently searched eight databases, including Web of Science, Cochrane Library, Embase, EBSCOhost, PubMed, China National Knowledge Infrastructure (CNKI), Wanfang, and VIP. Additionally, a manual search of reference lists was performed to supplement the database search. The search period covered from the establishment of each database to September 15, 2025. Randomized controlled trials (RCTs) investigating the effects of multicomponent exercise interventions on older adults with MCI were collected. Both subject terms and free-text terms were used in the search strategy. The English search terms included: Cognitive Dysfunction, Cognitive Disorder, Cognitive Impairment, Mild Cognitive Impairment, Cognitive Decline, Mental Deterioration, amnestic Mild Cognitive Impairment, MCI, Exercise, Physical Exercise, Aerobic Exercise, Isometric Exercise, Acute Exercise, Exercise Training, Physical Activity, Multicomponent Exercise, Multimodal Exercise, Combined Exercise, Mixed Exercise, and Randomized Controlled Trial.

### 2.2 Inclusion criteria

(1) The study population consisted of older adults with MCI, with no restrictions on sex, ethnicity, or nationality. (2) The intervention was multicomponent exercise, defined as a combination of two or more types of exercise (e.g., aerobic exercise, strength training, balance training, coordination training, flexibility training, etc.). The control group could receive usual care, health education, or similar interventions. (3) The primary outcome was overall cognitive function, assessed using standardized scales such as the Montreal Cognitive Assessment (MoCA) and the Mini-Mental State Examination (MMSE). Secondary outcomes included executive function and the concentration of BDNF. Executive function was evaluated using the following tests: working memory was assessed through the backward digit span test and working memory tasks; cognitive flexibility was measured using verbal fluency tests and the Trail Making Test (B/A ratio); and inhibitory control was evaluated using the interference condition completion time of the Stroop Color-Word Test. BDNF concentration was determined by collecting peripheral blood samples from participants and measuring BDNF levels. (4) Only RCTs were included.

### 2.3 Exclusion criteria

(1) Animal studies. (2) Review articles or conference papers. (3) Studies involving interventions other than exercise, such as cognitive therapy. (4) Studies with unavailable or incomplete data, even after contacting the authors for the original information.

### 2.4 Literature screening and data extraction

First, all bibliographic records retrieved from the databases were imported into EndNote reference management software. The software’s built-in “Find Duplicates” function was used to automatically identify and compare records based on key fields such as title, author, publication year, journal source, and DOI, thereby removing duplicate entries. Subsequently, the formal screening process was conducted independently and in parallel by two researchers. Each researcher reviewed the titles and abstracts of the remaining studies and made preliminary inclusion decisions according to the predefined inclusion and exclusion criteria. Full texts of studies deemed potentially eligible were then retrieved and examined in detail to confirm final inclusion. After independent screening and data extraction, the two researchers cross-checked and verified all results. Any disagreements were resolved through discussion with a third investigator. The extracted data included: (1) Basic study information—first author, publication year, country, participant age, and diagnostic source; (2) Study design and implementation characteristics—study type, sample size, intervention group details (exercise modality, exercise intensity, exercise duration per session, exercise frequency, and intervention duration), control group details, and reported adverse events; (3) Outcome measures—all relevant outcome data, including but not limited to primary outcomes (e.g., overall cognitive function scores) and secondary outcomes (e.g., scores of executive function subdomains), with corresponding means, standard deviations, and sample sizes at baseline and post-intervention time points.

### 2.5 Quality assessment

The methodological quality of the randomized controlled trials was assessed using the Physiotherapy Evidence Database (PEDro) scale. The evaluation was independently conducted by two researchers, and any discrepancies were resolved through discussion with a third investigator. The PEDro scale consists of 10 criteria, each scored as either “yes” or “no,” with a maximum total score of 10 points. Studies scoring 9–10 points were classified as high quality, 6–8 points as good quality, 4–5 points as fair quality, and below 4 points as low quality (Maher et al., 2003).

The quality of evidence for each outcome was assessed using the GRADE approach (Guyatt et al., 2008). Evidence was categorized into one of four levels: high, moderate, low, or very low. Two researchers independently performed the evidence grading. Any discrepancies were resolved through discussion until a consensus was reached, with the involvement of a third researcher if necessary.

### 2.6 Data analysis

All statistical analyses were performed using R software. To account for the dependency of effect sizes within studies, a three-level random-effects meta-analytic model was employed. This model partitions the variability in effect sizes into three levels: sampling variance (Level 1), within-study variance (Level 2), and between-study variance (Level 3). Standardized mean differences (SMD) and their variances were calculated using post-test means, standard deviations, and sample sizes from both the intervention and control groups. Effect sizes were estimated using Hedges’ *g* with 95% confidence intervals (CIs) via restricted maximum-likelihood (REML) estimation. According to Cohen’s conventions, *g* ≤ 0.20 was considered a small effect, 0.20–0.49 a small-to-medium effect, 0.50–0.79 a medium effect, and *g* ≥ 0.80 a large effect (Sheline et al., 1996). Likelihood ratio tests (LRTs) were used to test the statistical significance of the within-study (Level 2) and between-study (Level 3) variance components. Ninety-five percent prediction intervals (PIs) were calculated to determine the expected range of effect sizes in future comparable studies (Cui et al., 2024). Subgroup analyses were conducted using a mixed-effects model, with the statistical significance of between-group differences assessed via the Q-test. For each outcome, multiple moderator variables were examined separately in subgroup analyses. To control the risk of false positives due to multiple comparisons, the Bonferroni correction was applied to the tests of moderator variables for each outcome, with the corrected significance level set at *α*’ = 0.05/m, where m is the number of moderator tests performed for that outcome. A moderator effect was considered statistically significant only when *P* < *α*’ after correction. Subgroups with three or more effect sizes (*k* ≥ 3) were subjected to statistical inference, whereas those with two or fewer (*k* ≤ 2) were reported descriptively only. Statistical significance was defined as *p* < 0.05. Funnel plots and Egger’s regression test were used to assess publication bias.

## 3 Results

### 3.1 Literature search results

A total of 5,388 records were initially identified through database searches. After removing duplicates, 4,877 studies remained. Following title and abstract screening, 4,774 studies were excluded. The full texts of 103 studies were reviewed for eligibility, and finally, 10 randomized controlled trials were included in the meta-analysis, as shown in Figure 1.

**Figure 1:**
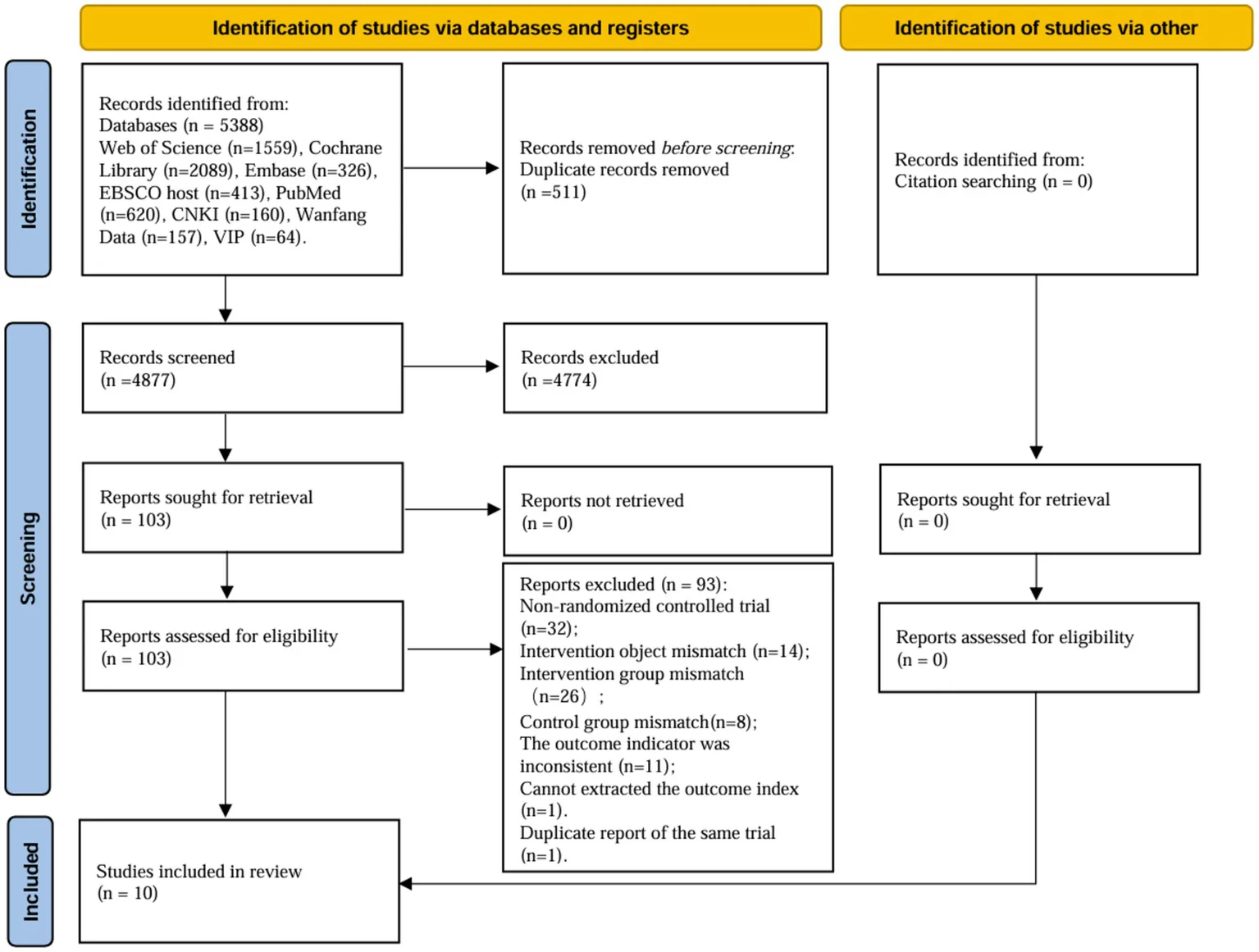
PRISMA flow diagram of study selection.

### 3.2 Characteristics of included studies

This meta-analysis ultimately included 10 randomized controlled trials (RCTs), all of which enrolled older adults with MCI, yielding a total pooled sample size of 623 participants. Participants were recruited from community screenings, hospitals, and nursing homes. The intervention consisted of multicomponent exercise, predominantly of moderate intensity, with exercise duration per session ranging from 30 to 90 min, exercise frequency of 2–5 sessions per week, and intervention duration of 8–48 weeks. Control groups received usual care or health education. Regarding safety, most studies explicitly reported no exercise-related adverse events. The primary outcome measures included global cognitive function, executive function, and BDNF levels. The detailed characteristics of the included studies are summarized in Table 1.

**Table 1:** Summary of the basic characteristics of included studies.

Study (author, year)	Country	Sample size (T/C)	Participant source	Sex (M/F)	Age (years, T/C)	Intervention group details	Intensity	Frequency (sessions/week)	Exercise duration per session (minutes)			Intervention duration (weeks)	Control group details	Adverse events	Outcome measures
Yin et al. (2023)	China	12/14	Community Screening	T:4/8; C:4/10	T:73.83 ± 7.51; C:74.77 ± 6.04	Aerobic Exercise + Strength Training + Flexibility Training + Balance Training	Moderate intensity, target heart rate 45–75% of maximum heart rate		3	60	20	Maintain usual lifestyle	No exercise-related adverse events	MOCA, MMSE, Digit Span Test - Backward, Verbal Fluency Test, Trail Making Test, BDNF	
Langoni et al. (2001)	Brazil	26/26	Community Screening	T:20/6; C:20/6	T:72.6 ± 7.8; C: 71.9 ± 7.9	Aerobic Exercise + Strength Training	Aerobic: 60–75% of maximum heart rate; Strength: progressive load		2	60	24	Maintain usual lifestyle	No exercise-related adverse events	MMSE	
Suzuki et al. (2012)	Japan	25/25	Community Screening	T:13/12; C:14/11	T:75.3 ± 7.5; C:76.8 ± 6.8	Aerobic Exercise + Strength Training + Balance Training	Aerobic: 60% of maximum heart rate		2	90	48	Health education	No exercise-related adverse events	MMSE, Letter Verbal Fluency Test, Category Verbal Fluency Test, Stroop Color-Word Test - Part III	
Suzuki et al. (2013)	Japan	50/50	Community Screening	T:25/25; C:26/24	T:74.8 ± 7.4; C:75.8 ± 6.1	Aerobic Exercise + Strength Training + Balance Training	Approximately 60% of maximum heart rate		2	90	24	Health education sessions twice	No exercise-related adverse events	MMSE, Alzheimer’s Disease Assessment Scale - Cognitive Subscale	
Bademli et al. (2019)	Turkey	30/30	Nursing Home	T:12/18; C:13/17	T: 72.24 ± 7.16; C:70.67 ± 8.34	Flexibility Training + Strength Training + Aerobic Exercise	Moderate intensity (3–6 METs)		4	80	20	Maintain usual lifestyle	No adverse events reported	MMSE	
Casas-Herrero et al. (2022)	Spain	88/100	Hospital	T:63/2;5 C:69/31	T:84.2 ± 4.8; C:84.0 ± 4.8	Strength Training + Balance Training + Flexibility Training + Aerobic Exercise	Moderate intensity (progressive load)		5		12	Routine outpatient care	No adverse events	MOCA	
Damirchi et al. (2018)	Iran	11/9	Yas Adult Day Care Center	T:0/11; C:0/9	T:68.81(3.68); C:69.11 (4.93)	Aerobic Exercise + Strength Training + Joint Mobility Training	Aerobic: 55–75% heart rate reserve (progressive); Strength: low intensity (RPE 13–15)		3	36 ~ 60	8	Maintain usual lifestyle	No adverse events reported	Working Memory Tests, BDNF	
Li et al. (2021)	China	42/42	Community Screening	T:15/27; C:18/24	>60	Aerobic Exercise + Strength Training + Balance Training + Coordination Training + Agility Training	RPE: 3–5/10 (low to moderate intensity)		5	30	24	Routine community health guidance	No adverse events reported	MMSE, MOCA	
Yu et al. (2022)	China	12/12	Community Screening	T: 4/8; C: 2/10	T: 67.2 ± 6.8; C: 67.6 ± 8.1	Flexibility Training + Strength Training + Aerobic Exercise	Moderate intensity (RPE ≈ 13)		3	60	24	Maintain usual lifestyle	No adverse events	MOCA, Digit Span Backward - Length, Verbal Fluency Test, Trail Making Test Part B/A Ratio Score, Victoria Stroop Test - Interference Condition	
De Oliveira Silva et al. (2019)	Brazil	7/12	Hospital	T:1/6; C:7/5	T: 71.85 ± 5.69; C: 78.20 ± 5.26	Balance Training + Aerobic Exercise + Strength Training + Flexibility Training	Moderate intensity (70% VO₂max or 80% HRmax)		2	60	12	Clinical follow-up without exercise training	No exercise-related adverse events	MMSE, Verbal Fluency, Stroop Test - Interference Condition	

T/C: intervention/control; M/F: male/female; min: minutes; RPE: rating of perceived exertion; MoCA: Montreal cognitive assessment; MMSE: mini-mental state examination; BDNF: brain-derived neurotrophic factor.

### 3.3 Methodological quality assessment of included studies

As presented in Table 2, the methodological quality scores of the 10 included studies ranged from 4 to 7, with a mean score of 6.0, indicating an acceptable overall quality. All studies reported random allocation, baseline similarity between groups, statistical comparisons between groups, and point measures with variability data. None of the studies implemented participant or therapist blinding. Two studies reported allocation concealment (Suzuki et al., 2013; Yu et al., 2022). Seven studies implemented assessor blinding (Langoni et al., 2001; Suzuki et al., 2012; Suzuki et al., 2013; Casas-Herrero et al., 2022; Li et al., 2021; Yu et al., 2022; De Oliveira Silva et al., 2019). Two studies had participation rates below 85% (Casas-Herrero et al., 2022; Damirchi et al., 2018). Three studies applied intention-to-treat (ITT) analysis (Suzuki et al., 2012; Bademli et al., 2019; Casas-Herrero et al., 2022).

**Table 2:** Methodological quality assessment of included studies.

Study (author, year)	Random allocation	Allocation concealment	Baseline similarity	Participant blinding	Therapist blinding	Assessor blinding	Adequate follow-up (>85% participation)	Intention-to-treat analysis	Between-group statistical comparisons	Point measures and variability data	Total score
Yin et al. (2023)	1	0	1	0	0	0	1	0	1	1	5
Langoni et al. (2001)	1	0	1	0	0	1	1	0	1	1	6
Suzuki et al. (2012)	1	0	1	0	0	1	1	1	1	1	7
Suzuki et al. (2013)	1	1	1	0	0	1	1	0	1	1	7
Bademli et al. (2019)	1	0	1	0	0	0	1	1	1	1	6
Casas-Herrero et al. (2022)	1	0	1	0	0	1	0	1	1	1	6
Damirchi et al. (2018)	1	0	1	0	0	0	0	0	1	1	4
Li et al. (2021)	1	0	1	0	0	1	1	0	1	1	6
Yu et al. (2022)	1	1	1	0	0	1	1	0	1	1	7
De Oliveira Silva et al. (2019)	1	0	1	0	0	1	1	0	1	1	6

### 3.4 Meta-analysis results

#### 3.4.1 Effects of multicomponent exercise on global cognitive function

A total of 10 studies with 12 effect sizes were included to examine the effects of multicomponent exercise on global cognitive function in older adults with MCI (Figure 2). Both the three-level meta-analysis (*g* = 1.063, 95% CI [0.355, 1.771], *p* = 0.007, 95% PI [−1.141, 3.267]) and the two-level meta-analysis (*g* = 1.068, 95% CI [0.426, 1.710], *p* = 0.004, 95% PI [−1.132, 3.268]) yielded significant overall effects, indicating that multicomponent exercise significantly improves global cognitive function in older adults with MCI.

**Figure 2:**
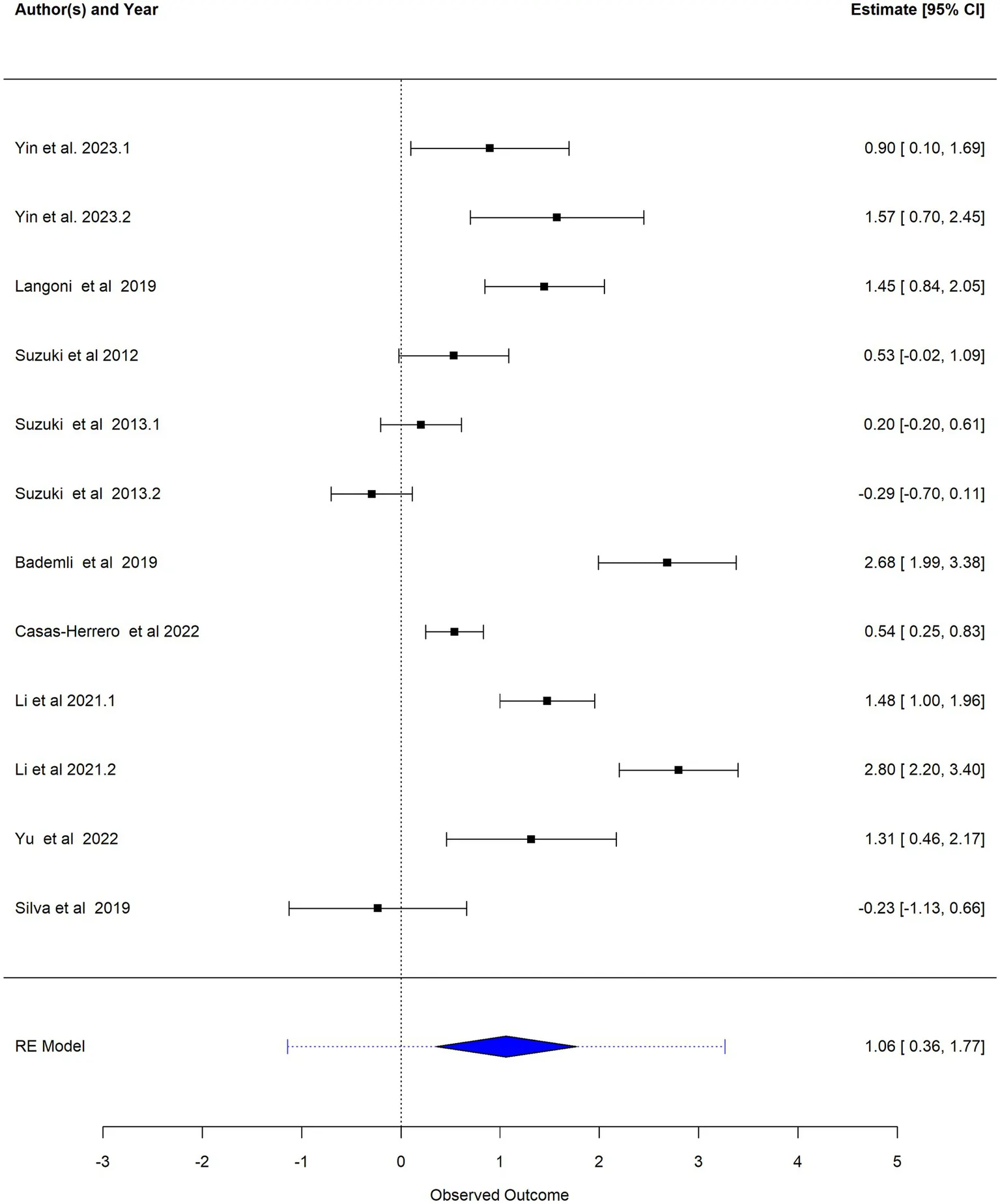
Forest plot of the effects of multicomponent exercise on global cognitive function in older adults with mild cognitive impairment.

Influence analysis was performed to examine whether any outliers influenced the meta-analytic results. As shown in Figure 3, no outliers requiring exclusion were identified, indicating that the meta-analytic findings are robust and reliable, and are not significantly affected by any single study.

**Figure 3:**
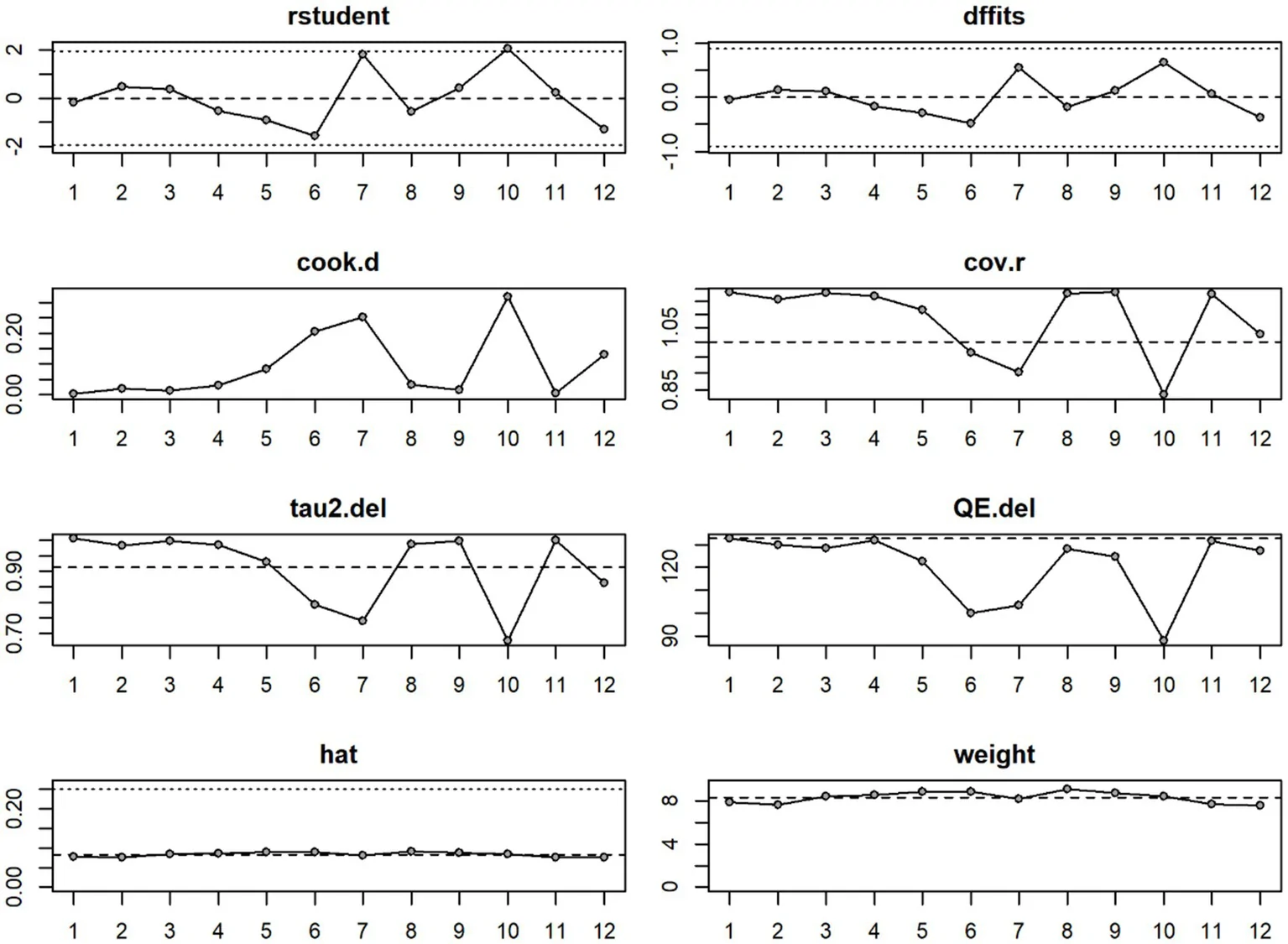
Influence analysis of global cognitive function.

Heterogeneity testing revealed significant heterogeneity across studies [Q(df = 11) = 132.816, *p* < 0.0001]. Of the total variance, sampling variance (Level 1) accounted for 7.63%, within-study variance (Level 2) for 31.04%, and between-study variance (Level 3) for 61.33%, indicating that between-study heterogeneity was the primary source of total variability. This finding justifies the use of a random-effects model and the three-level meta-analytic approach, and warrants subsequent moderator analyses to explore the sources of heterogeneity.

#### 3.4.2 Effects of multicomponent exercise on executive function

A total of 5 studies with 13 effect sizes were included to examine the effects of multicomponent exercise on executive function in older adults with MCI. Both the three-level meta-analysis (*g* = 0.468, 95% CI [0.093, 0.844], *p* = 0.0188, 95% PI [−0.424, 1.360]) and the two-level meta-analysis (*g* = 0.483, 95% CI [0.158, 0.808], *p* = 0.0072, 95% PI [−0.394, 1.359]) yielded significant overall effects, indicating that multicomponent exercise significantly improves executive function in older adults with MCI.

Influence analysis was performed to examine whether any outliers influenced the meta-analytic results, which identified one outlier (Figure 4). After excluding this outlier, the meta-analysis was re-estimated based on the remaining 12 effect sizes. The results of the three-level meta-analysis are presented in Figure 5 (*g* = 0.372, 95% CI [0.140, 0.604], *p* = 0.0047, 95% PI [0.140, 0.604]). The two-level meta-analysis yielded consistent results (*g* = 0.372, 95% CI [0.140, 0.604], *p* = 0.0047, 95% PI [0.140, 0.604]), with overall effects remaining significant. To avoid potential bias from this outlier, it was excluded from all subsequent analyses.

**Figure 4:**
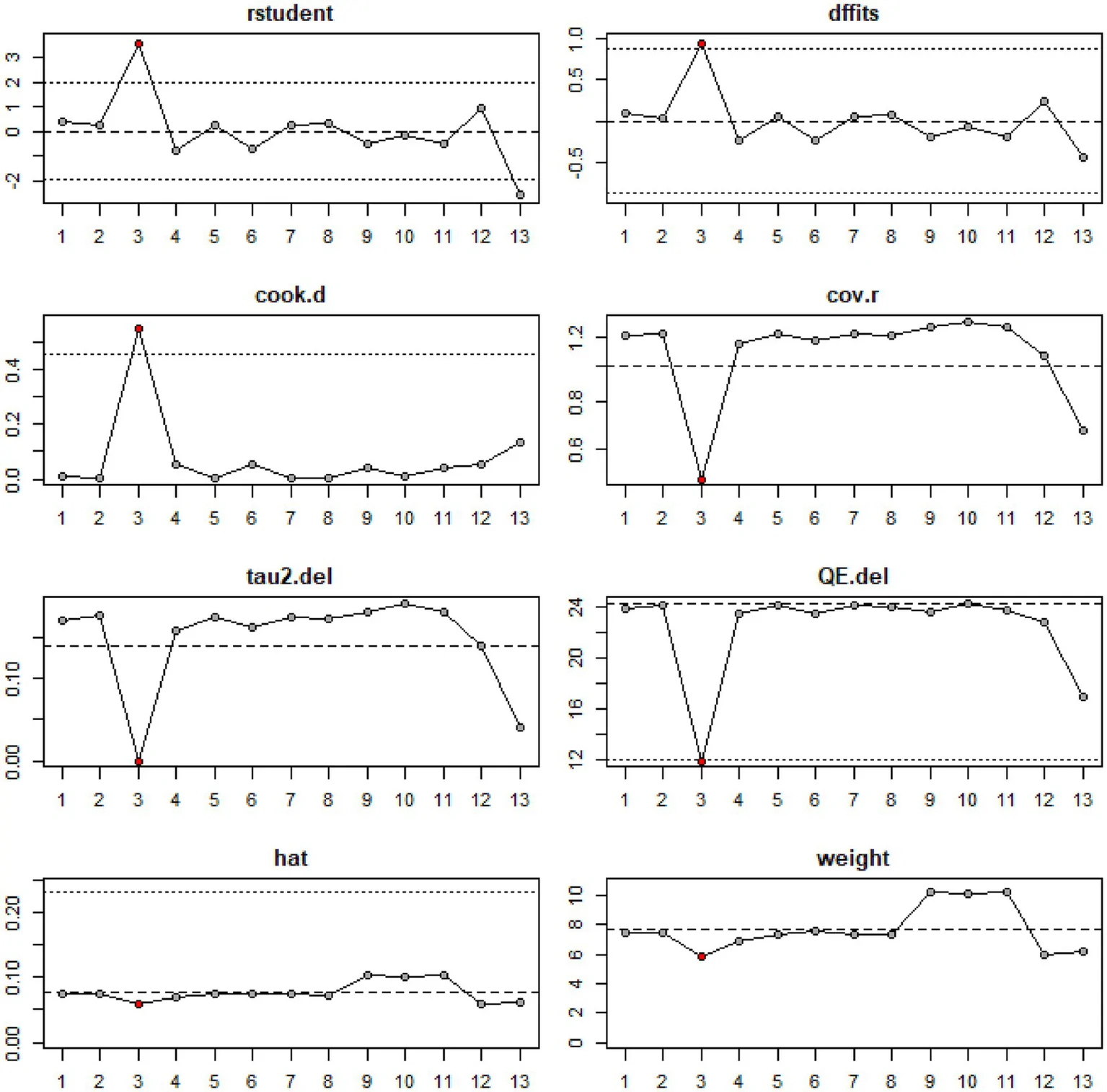
Influence analysis of executive function.

**Figure 5:**
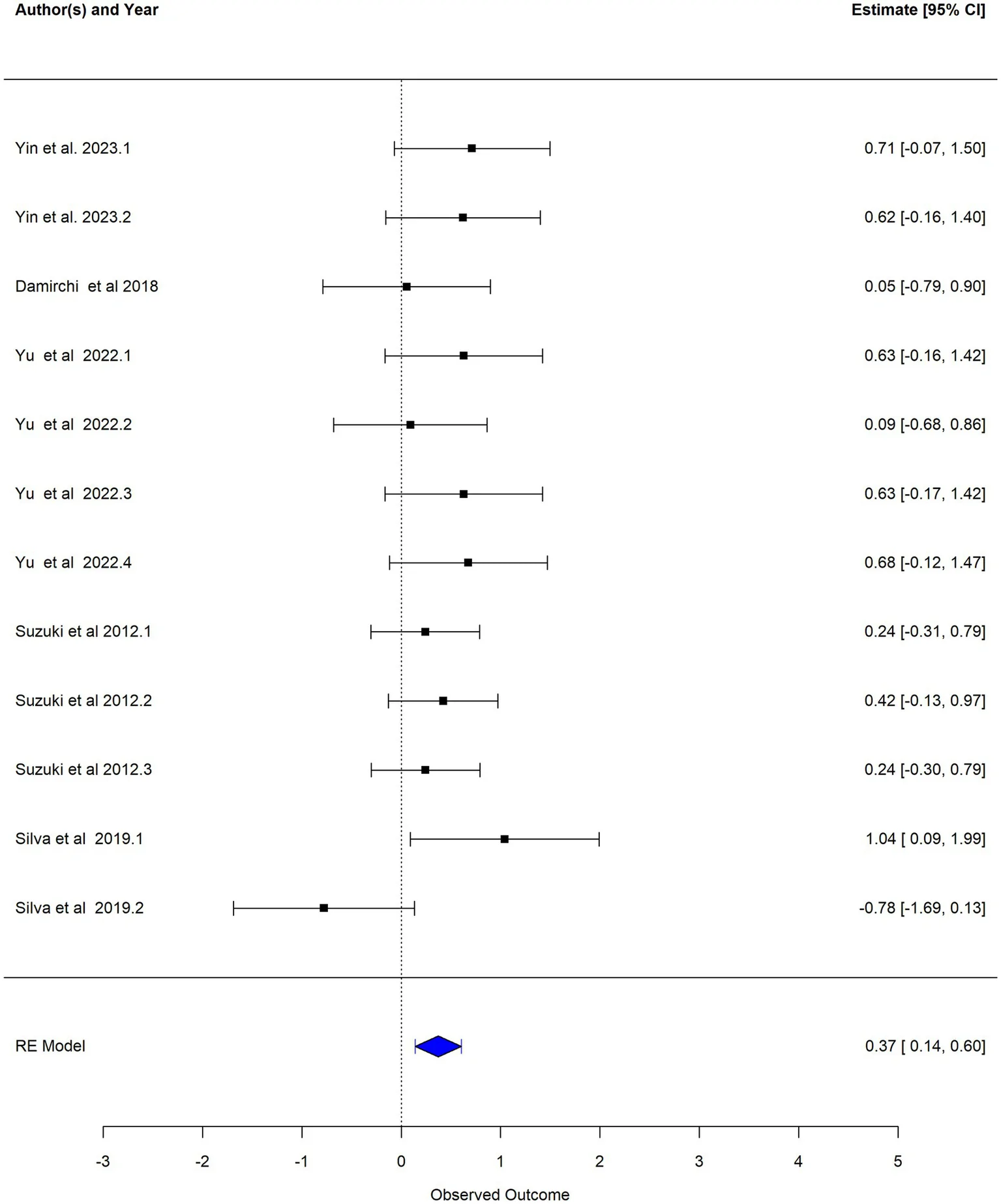
Forest plot of the effects of multicomponent exercise on executive function in older adults with mild cognitive impairment.

Heterogeneity testing based on the remaining 12 effect sizes yielded Q(df = 11) = 11.994, *p* = 0.364, and *I*^2^ = 0.00%, indicating no significant heterogeneity across studies. Further variance decomposition revealed that sampling variance (Level 1) accounted for 100%, while within-study (Level 2) and between-study (Level 3) variances both approached 0%, indicating that the variability in effect sizes stemmed primarily from sampling error rather than from within- or between-study differences.

#### 3.4.3 Effects of multicomponent exercise on BDNF

Two studies with two effect sizes reported the effects of multicomponent exercise on BDNF levels in older adults with MCI. Yin et al. (2023) reported a significant increase in serum BDNF levels in older adults with MCI following multicomponent exercise (intervention group change: 9.29 ± 3.48 ng/mL; control group change: −2.50 ± 4.25 ng/mL), with a statistically significant between-group difference (*p* < 0.001). By contrast, Damirchi et al. (2018) reported no significant change in serum BDNF levels following an 8-week aerobic exercise intervention in older adults with MCI (intervention group change: −45.05 ± 511.76 pg./mL; control group change: −189.70 ± 472.17 pg./mL). The direction and magnitude of the effects differed considerably between the two studies.

#### 3.4.4 Exploratory subgroup analyses

To explore the potential sources of heterogeneity, exploratory subgroup analyses were conducted on exercise modality, session duration, exercise frequency, intervention duration, and measurement tools. For session duration, Casas-Herrero et al. (2022) was excluded owing to missing data. Subgroup analysis results are presented in Table 3.

**Table 3:** Results of exploratory subgroup analyses.

	Subgroup	Number of effect sizes	*g* (95% CI)	*p*	*F* (df1, df2), *p*
Outcome global cognitive function	Exercise modality				—
	Aerobic + strength + balance + coordination + agility training	2	2.109 (0.816, 3.402)	0.006	
	Aerobic + strength + flexibility + balance training	4	0.645 (−0.268, 1.558)	0.139	
	Aerobic + strength + flexibility training	2	2.039 (0.772, 3.307)	0.007	
	Aerobic + strength + balance training	3	0.164 (−0.841, 1.168)	0.711	
	Aerobic + strength training	1	1.448 (−0.247, 3.142)	0.083	
	Exercise duration per session				*F*(1, 9) = 0.127, *p* = 0.730
	30 ~ 60 min	7	1.236 (0.151, 2.321)	0.030	
	80–90 min	4	0.959 (−0.421, 2.339)	0.150	
	Exercise frequency				*F*(1, 10) = 3.4213, *p* = 0.094
	2–3 sessions per week	8	0.685 (−0.088, 1.457)	0.077	
	4–5 sessions per week	4	1.772 (0.714, 2.830)	0.004	
	Intervention duration				*F*(1, 10) = 0.0002, *p* = 0.988
	12–20 weeks	5	1.070 (−0.095, 2.234)	0.068	
	24–48 weeks	7	1.059 (0.044, 2.073)	0.042	
	Measurement tools				—
	MOCA	4	1.337 (0.235, 2.439)	0.023	
	MMSE	7	1.054 (0.214, 1.895)	0.020	
	ADAS-cog	1	−0.036 (−1.961, 1.889)	0.968	
Executive function	Dimension				*F*(2, 9) = 0.769, *p* = 0.492
	Working memory	3	0.484 (−0.053, 1.021)	0.072	
	Cognitive flexibility	6	0.437 (0.112, 0.761)	0.014	
	Inhibitory control	3	0.154 (−0.313, 0.621)	0.474	

All subgroup analyses were exploratory in nature, and the results should be interpreted with caution. Interaction tests were performed for statistical inference only on categories with three or more effect sizes (*k* ≥ 3) within each subgroup. For subgroups containing categories with *k* ≤ 2 (i.e., exercise modality and measurement tools), the interaction *p* value is denoted as “—”.

For global cognitive function, session duration [*F*(1, 9) = 0.127, *p* = 0.730], exercise frequency (*F*(1, 10) = 3.421, *p* = 0.094), and intervention duration [*F*(1, 10) = 0.0002, *p* = 0.988] all showed no significant moderating effects. For exercise modality, interaction tests were not performed owing to the limited number of effect sizes in certain categories (*k* ≤ 2), and results across groups were presented descriptively. Specifically, the combinations of “aerobic + strength + balance + coordination + agility” training (*k* = 2, *g* = 2.109, 95% CI [0.816, 3.402], *p* = 0.006) and “aerobic + strength + flexibility” training (*k* = 2, *g* = 2.039, 95% CI [0.772, 3.307], *p* = 0.007) both yielded significant improvements in global cognitive function, whereas other modalities did not reach statistical significance. For measurement tools, the ADAS-Cog was included in only one effect size (*g* = −0.025, 95% CI [−2.088, 2.039]), and the result was reported descriptively.

For executive function (Table 3), subgroup analyses based on the 12 effect sizes after outlier exclusion revealed that no significant moderating effects were observed across executive function subdomains. Specifically, cognitive flexibility was significantly improved (*g* = 0.437, 95% CI [0.112, 0.761], *p* = 0.014), whereas working memory and inhibitory control did not reach statistical significance.

#### 3.4.5 Publication bias

To assess publication bias, funnel plot visual inspection, Egger’s regression test, and the nonparametric trim-and-fill method were employed. As shown in Figure 6(1), the funnel plot for global cognitive function exhibited a roughly symmetrical distribution of study points. Egger’s regression test indicated no significant asymmetry (QM = 0.926, *p* = 0.359). The trim-and-fill analysis revealed that no studies were imputed on either side (imputed studies = 0), and the original effect size (*g* = 1.046) was identical to the adjusted effect size (*g* = 1.046). Taken together, these findings suggest no substantial publication bias, indicating that the results are robust and reliable.

**Figure 6:**
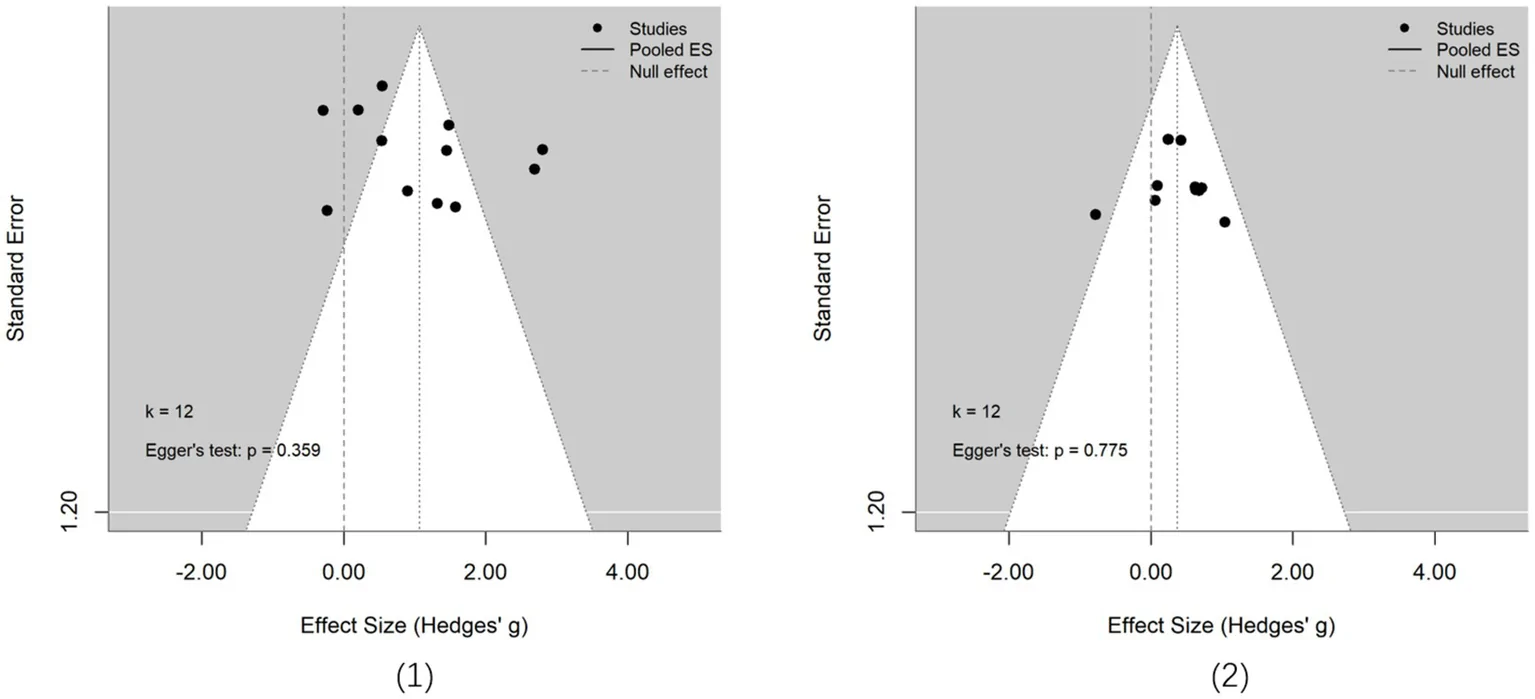
Funnel plots.

As shown in Figure 6(2), the funnel plot for executive function—based on the 12 effect sizes after outlier exclusion—exhibited a roughly symmetrical distribution. Egger’s regression test indicated no significant asymmetry (QM = 0.086, *p* = 0.776). The trim-and-fill analysis showed that no studies were imputed on the left side (imputed studies = 0), with the original effect size (*g* = 0.372) identical to the left-adjusted effect size (*g* = 0.372); on the right side, one study was imputed, yielding an adjusted effect size of *g* = 0.393, which differed minimally from the original effect size (*g* = 0.372). Taken together, these findings suggest no substantial publication bias, indicating that the results are robust and reliable.

#### 3.4.6 Quality of evidence assessment

As assessed by the GRADE system (Figure 7), the quality of evidence was rated as low for global cognitive function and as moderate for executive function. The downgrading was primarily attributable to the lack of allocation concealment and incomplete blinding in most included studies, as well as substantial heterogeneity.

**Figure 7:**
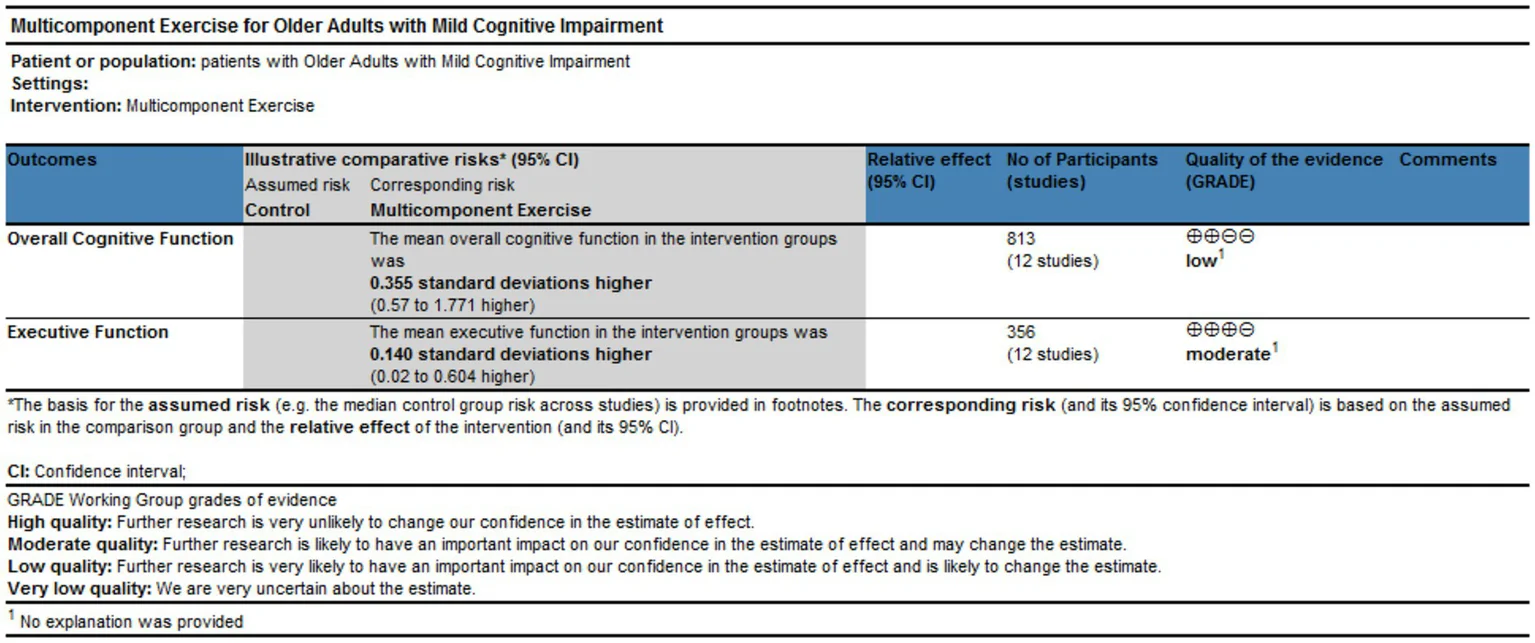
GRADE assessment of the certainty of evidence.

## 4 Discussion

The findings of this study indicate that multicomponent exercise improves global cognitive function and executive function in older adults with MCI, whereas its effects on BDNF levels remain inconclusive. Exploratory subgroup analyses did not identify any statistically robust effect modifiers, as none of the interaction tests reached statistical significance.

The methodological quality scores of the included studies ranged from 4 to 7, with a mean of 6.0, indicating generally good quality. However, several limitations should be noted: most studies did not implement allocation concealment, and none of the studies were able to blind participants or personnel owing to the nature of the exercise interventions, which may have influenced post-test outcomes. Publication bias tests for global cognitive function and executive function indicated no evidence of publication bias. No significant downgrading was identified for indirectness in the quality of evidence assessment. The meta-analysis revealed substantial heterogeneity for global cognitive function, although the current exploratory subgroup analyses did not clarify its specific sources. By contrast, no notable heterogeneity was observed across studies for executive function. Consequently, the quality of evidence was rated as low for global cognitive function and as moderate for executive function. For BDNF, no meta-analysis was performed owing to the limited number of included studies; therefore, publication bias tests and GRADE assessment were not conducted.

The results of this study indicate that multicomponent exercise significantly improves global cognitive function in older adults with MCI, which is consistent with previous findings (Wang et al., 2020; Akalp et al., 2024). Multicomponent exercise may therefore serve as an effective non-pharmacological intervention for improving cognitive function in this population. However, substantial heterogeneity was observed across studies, with variance decomposition identifying between-study heterogeneity as the primary source of total variability, suggesting that the intervention effects may be moderated by multiple factors. Nevertheless, the current exploratory subgroup analyses did not clarify the primary sources of this heterogeneity. For exercise modality, interaction tests were not performed owing to the limited number of effect sizes in certain categories (*k* ≤ 2), and results across groups were presented descriptively. Among these, the combinations comprising aerobic, strength, balance, coordination, and agility training, as well as the combination of aerobic, strength, and flexibility training, yielded large effect sizes. However, owing to the limited number of effect sizes, these findings should be interpreted with caution and definitive conclusions cannot be drawn. The potential mechanisms may involve synergistic effects through distinct physiological and neural pathways: aerobic exercise enhances cerebral blood flow and neurotrophic factor expression; strength training improves metabolic status and modulates neuroendocrine function; and flexibility, balance, and coordination training contribute to enhanced neural network connectivity and flexible allocation of cognitive resources. Collectively, these mechanisms act upon different cognitive domains, thereby facilitating the optimization of global cognitive function through multimodal synergy (Dhahbi et al., 2025; Liao et al., 2026; Wang et al., 2025). With respect to session duration, interventions of 30–60 min significantly improved global cognitive function, whereas those of 80–90 min did not reach statistical significance. For exercise frequency, interventions of 4–5 sessions per week yielded significant effects, whereas those of 2–3 sessions per week did not reach statistical significance. Regarding intervention duration, interventions of 24–48 weeks showed significant effects, whereas those of 12–20 weeks did not reach statistical significance. In summary, the current subgroup analyses did not identify any statistically robust effect modifiers. The above findings are exploratory in nature and warrant confirmation in future studies.

The present study found that multicomponent exercise significantly improved executive function in older adults with MCI. Subgroup analyses revealed no significant moderating effects across executive function subdomains; however, cognitive flexibility was significantly enhanced, whereas working memory and inhibitory control did not reach statistical significance. This discrepancy may be attributable to the distinct neural mechanisms underlying different executive function subdomains and their differential responsiveness to exercise interventions. Cognitive flexibility is primarily supported by neural circuits connecting the prefrontal cortex, parietal lobes, and striatum. Multicomponent exercise may exert its beneficial effects by enhancing neuroplasticity and functional connectivity within these regions. By contrast, working memory and inhibitory control may involve more extensive frontotemporal networks and hippocampo-prefrontal projections, the improvement of which may require longer or higher-intensity intervention stimuli (Diamond, 2013; Barbey et al., 2013; Aron et al., 2014). Notably, given the limited sample size in these subgroup analyses, the results may be subject to insufficient statistical power, which to some extent weakens the robustness of the current conclusions. Future studies with larger samples are warranted to validate these findings. Regarding BDNF, only two studies were included in this review. Owing to the very limited number of effect sizes, no meta-analysis was performed, and the results were summarized narratively. Yin et al. (2023) reported a significant increase in serum BDNF levels in older adults with MCI following multicomponent exercise, whereas Damirchi et al. (2018) reported no significant change following an 8-week aerobic exercise intervention. The direction and magnitude of the effects differed considerably between the two studies, which may be attributable to differences in intervention protocols and methodological heterogeneity in BDNF assays. Although BDNF plays an important role in cognitive protection as a neurotrophic factor (Huang and Reichardt, 2001), the current evidence is insufficient to draw definitive conclusions regarding the effects of multicomponent exercise on BDNF levels in older adults with MCI. Future studies with standardized assay protocols and higher methodological quality are warranted to further validate these findings.

Several limitations of this study should be acknowledged. First, the small number of included studies on BDNF precluded meta-analysis, and the limited number of studies on executive function constrained the feasibility of subgroup analyses. Second, heterogeneity across studies in exercise protocols, assessment tools, and sample characteristics may have contributed to the substantial heterogeneity observed. In addition, the subgroup analyses involved multiple comparisons, and all subgroup findings are exploratory in nature and warrant confirmation in future studies. Future studies should build upon larger sample sizes, further optimize multicomponent exercise protocols, and identify the optimal exercise dose and component combinations. Standardized cognitive assessment tools and unified biospecimen assay procedures should be adopted, and greater emphasis should be placed on exploring potential biomarkers (e.*g*., BDNF) to elucidate the underlying mechanisms by which exercise improves cognitive function.

## 5 Conclusion

Multicomponent exercise exerts positive effects on global cognitive function and executive function in older adults with MCI, whereas its effects on BDNF remain inconclusive. Future studies with more rigorous designs and adequate sample sizes are warranted to promote the personalized application of multicomponent exercise in the MCI population.

## Data Availability

The original contributions presented in the study are included in the article/Supplementary material. Further inquiries can be directed to the corresponding author.
